# Antiviral therapy in shrimp through plant virus VLP containing VP28 dsRNA against WSSV

**DOI:** 10.3762/bjoc.17.95

**Published:** 2021-06-01

**Authors:** Santiago Ramos-Carreño, Ivone Giffard-Mena, Jose N Zamudio-Ocadiz, Alfredo Nuñez-Rivera, Ricardo Valencia-Yañez, Jaime Ruiz-Garcia, Maria Teresa Viana, Ruben D Cadena-Nava

**Affiliations:** 1 Facultad de Ciencias Marinas, Universidad Autónoma de Baja California (UABC), Carretera Transpeninsular Ensenada-Tijuana No. 3917, Colonia Playitas, C.P. 22860 Ensenada, B.C., México; 2 Centro de Nanociencias y Nanotecnología, Universidad Nacional Autónoma de México (UNAM). Km 107 Carretera Tijuana-Ensenada, Col. Pedregal Playitas, C.P. 22860 Ensenada, B.C., México; 3 Centro de Investigación Científica y de Educación Superior de Ensenada, Baja California, (CICESE), Carretera Ensenada - Tijuana No. 3918, Zona Playitas, C.P. 22860, Ensenada, B.C., México; 4 Instituto de Física, Universidad Autónoma de San Luis Potosí, Álvaro Obregón 64, San Luis Potosí 78000, México; 5 Instituto de Investigaciones Oceanológicas, Universidad Autónoma de Baja California (UABC), Carretera Transpeninsular Ensenada-Tijuana No. 3917, Colonia Playitas, C.P. 22860 Ensenada, B.C., México

**Keywords:** antiviral therapy, CCMV, oral administration, *P. vannamei*, plant VLPs, RNAi, VP28, white spot syndrome virus

## Abstract

The white spot syndrome virus (WSSV), currently affecting cultured shrimp, causes substantial economic losses to the worldwide shrimp industry. An antiviral therapy using double-stranded RNA interference (dsRNAi) by intramuscular injection (IM) has proven the most effective shrimp protection against WSSV. However, IM treatment is still not viable for shrimp farms. The challenge is to develop an efficient oral delivery system that manages to avoid the degradation of antiviral RNA molecules. The present work demonstrates that VLPs (virus-like particles) allow efficient delivery of dsRNAi as antiviral therapy in shrimp. In particular, VLPs derived from a virus that infects plants, such as cowpea chlorotic mottle virus (CCMV), in which the capsid protein (CP) encapsidates the dsRNA of 563 bp, are shown to silence the WSSV glycoprotein VP28 (dsRNAvp28). In experimental challenges in vivo, the VLPs- dsRNAvp28 protect shrimp against WSSV up to 40% by oral administration and 100% by IM. The novel research demonstrates that plant VLPs, which avoid zoonosis, can be applied to pathogen control in shrimp and also other organisms, widening the application window in nanomedicine.

## Introduction

The white spot syndrome virus (WSSV) is recognized as one of the most severe epidemic pathogens of shrimp, causing severe economic losses to shrimp aquaculture. More than three decades ago Chou et al. [1] first described the emergence of this pathogen and since then, rapidly, it has spread globally [2–3]. The aquaculture industry still suffers productive and economic impacts from the outbreak, causing up to 100% mortality in shrimp farms within 3 to 10 days [1,4]. The rapid propagation and susceptibility of WSSV infection in several species, particularly the white shrimp *Penaeus vannamei* [5–6], have sparked intense research for its prevention and control [7].

So far several strategies have been reported to control the WSSV, including activation of the immune system, DNA vaccines, herbal extracts, and RNA interference (RNAi) [8–9]. Among them, the RNAi technology has shown great potential to protect shrimp against the WSSV in some lab-scale experiments [10–11]. The RNAi mechanism comprises a set of cellular processes of posttranscriptional gene silencing that begins with administering the double-stranded RNA (dsRNA). It concludes with a specific gene silencing based on sequence homology between the digested fragments of the dsRNA and the gene of interest [12–16]. The antiviral response of RNAi is triggered by double-stranded RNA (dsRNA) to block the synthesis of a specific viral protein, in the case of WSSV the targets being the structural proteins VP19, VP24, VP26, and VP28, as they are involved in cell recognition, virus entry, binding and assembly of the virion. Previous studies have shown that silencing these structural proteins in WSSV challenge assays, increases shrimp survival [10–11,17–21]. The VP28 glycoprotein plays an important role in systemic infection by interacting with cell membrane proteins, and it is one of the most abundant proteins along with VP26 (≈60%) in the external WSSV surface [21–22].

The RNAi trials using an intramuscular injection (IM) have shown that VP28 glycoprotein is the target of choice to block WSSV infection in shrimp [14,23–24]. However, RNAi intramuscular (IM) administration is limited to lab-scale experiments since its use is not yet viable for applications on a large scale, as found in salmon farms [25]. The naked RNA degrades quickly when supplied in feed [26–27], either due to feed processing or the digestion process [20]. The challenge is to develop a treatment through the oral route [11,28] instead of IM, yet one in which the RNA is nonetheless is protected.

One solution is a nanocarrier [11,27,29] to protect, stabilize and maintain the integrity of the RNAi in the environment [14]. Recently, dsRNA has been integrated into nanovehicles such as non-virulent capsids or virus-like particles (VLPs) [30–32] lacking the viral genome. Their small size (20–140 nm), allows them to permeate the cell membranes without causing toxicity or immune response in the treated organisms [30,32–36]. In particular, the VLPs derived from plant viruses are attractive, since any zoonotic possibility is eliminated, being biocompatible and biodegradable [34,36–37]. Its structure presents advantages over other synthetic nanomaterials, as it is simple and easy to purify for large scale production [34,37–38].

The plant virus cowpea chlorotic mottle virus (CCMV) has been extensively studied and characterized, due to its potential applications in nanomedicine [33,36,39–41]. Native CCMV has a positive single-stranded RNA. It is a Bromoviridae family member that infects cowpea *(Vigna unguiculata)* plants*.* The CCMV VLPs with heterologous RNA has already been in-vitro synthesized [32,42], being RNases resistant, and can release cargo in the cytoplasm of mammalian cells [32–33,43].

This work aims to evaluate the efficacy of CCMV VLP-VP28 dsRNA (VLP-dsRNAvp28) delivery against WSSV, by oral administration to shrimp through commercial feed pellets. Through in vivo bioassays, the antiviral efficacy of VLPs is assessed by intramuscular injection and per os, in *Penaeus vannamei* infected with WSSV.

To our knowledge, this is the first report where an oral VLPs are administered to treat infected shrimp against viruses. This is a novel technique in aquaculture.

## Materials and Methods

**dsRNAvp28*.*** The VP28 dsRNA (dsRNAvp28) was generated based on the VP28 sequence of WSSV (GenBank: EU931451.1) [44]. The sequence is shown in Supporting Information File 1, Table ST1. The dsRNAvp28 was purchased from groRNA/Genolution company (South Korea).

**CCMV capsid protein purification.** The plant virus CCMV was produced in California cowpea plants (*Vigna ungiculata*). The plants were mechanically inoculated with a solution containing the virus. After two weeks, the infected leaves were collected and ground in a virus extraction buffer (0.5 M sodium acetate, 0.08 M magnesium acetate, pH 4.5) using a kitchen blender. The obtained homogeneous extract was filtered through a cheesecloth to remove solid material. Then the homogenate was mixed with a half-volume of chloroform and centrifuged at 15,000 rpm for 15 min using a JA-14 rotor (Beckman Coulter, USA). After that, the supernatant was recovered and stirred for at least 3 h. Then the sample was layered on a 10% sucrose cushion and ultracentrifuged for 2 hours at 30,000 rpm using an SW-32Ti rotor (Beckman Coulter, USA). Later, the supernatant was discarded and the pellets were resuspended with a virus suspension buffer (50 mM sodium acetate, 8 mM magnesium acetate, pH 4.5). The solution was ultracentrifuged through a sucrose gradient at 30,000 rpm for 2 hours, at 4 °C. The virus was recovered from the blue band, and the sucrose was removed by ultracentrifugation. The pellets were resuspended in virus suspension buffer (50 mM sodium acetate, 8 mM magnesium acetate, pH 4.5). All the procedure was done at 4 °C. The virus’s concentration and purity were determined by UV–vis spectrophotometry, and the virus aliquots were kept at −80 °C.

The protein purification was performed according to a previously described protocol [40]. Briefly, the CCMV was dialyzed in a disassembly buffer (0.5 M CaCl_2_, 50 mM Tris, 1 mM EDTA, 1 mM DTT, 0.5 mM PMSF, pH 7.5) at 4 °C for 24 h. Then, the sample was ultracentrifuged at 50,000 rpm for 510 min at 4 °C, using a Beckman Type 90 Ti rotor. The pellet was discarded, and the supernatant containing the capsid protein (CP) was recovered. Later, the CP was dialyzed against a buffer (1 M NaCl, 20 mM Tris, 1 mM EDTA, pH 7.2) overnight. The protein concentration and purity were determined by UV–vis spectrophotometry; only CP samples with the wavelength ratio 280/260 ≥ 1.5 were used for the VLPs assembling. SDS-PAGE was used to verify the integrity of the capsid protein.

**In vitro assembly of VLPs-dsRNAvp28*.*** Dissociated CCMV CP and dsRNAvp28 were mixed in a mass ratio of 6:1 (CP/dsRNA) and dialyzed overnight against RNA assembly buffer (50 mM NaCl, 10 mM KCl, 5 mM MgCl_2_, 50 mM Tris-HCl, pH 7.2) at 4 °C. The samples were acidified by dialysis in virus suspension buffer (50 mM sodium acetate, 8 mM magnesium acetate, pH 4.5) for at least 4 hours. Then, to disrupt the empty capsids, the sample was dialyzed against an RNA assembly buffer. The VLP-dsRNAvp28 was then purified and concentrated by ultrafiltration with reassembly buffer using a 100 kDa Amicon centrifuge filter (0.5 mL, Millipore) at 8,000*g* for 15 min, and the step was repeated at least three times.

VLPs assembly products were analyzed by gel electrophoresis mobility shift assay (EMSA) in native agarose gel at 1%. The electrophoresis was run in a horizontal agarose gel system (FBSB710 Fisher Scientific) for 4 h at 50 volts (virus buffer), 4 °C and then, the gel was stained with ethidium bromide. The image was captured using a documentation system (MS Major Science).

**Transmission electron microscopy analysis of VLPs**. 6 µL of VLP-dsRNAvp28 from the assembly stock solution was placed onto a carbon-coated grid (400 mesh Cu, Ted Pella) for 2.5 min. The excess solution was removed with a Whatman filter paper, and the sample was stained with 6 µL of 2% uranyl acetate for 1 min. The samples were analyzed with a JEOL JEM-2010 transmission electron microscope equipped with a digital camera operated at 200 keV. The size of the VLPs was measured using the ImageJ (U.S. NIH) software from digital recorded TEM images.

**Shrimp and rearing conditions. ***P. vannamei* postlarvae (PL) were grown in 2,500 L circular tanks containing seawater (34 ppt salinity) at 28 ± 1 °C, oxygen > 5.0 mg/L, pH 7.6 ± 0.16 and ammonium < 0.5 mg/L. The postlarvae were fed a commercial diet (Natural Force 35^®^ VIMIFOS, Mexico) at 5% of the total biomass thrice a day. The seawater was filtered (10.5 and 5 µm sediment water filters, respectively), exposed to UV and aerated before use. Forty percent of water was replaced every three days to collect food waste and feces.

Once the PL reached a juvenile stage, they were transferred into 12 L aquariums. Each aquarium was equipped with a filter and a heating system (Titanium Heater HMO-200, JSK). The shrimp were immersed in a 0.002% formaldehyde solution in seawater for 30 min before transferring them to the aquariums to remove any fouling present. Six shrimp were placed per aquarium, containing seawater of 34 ppt at 28 ± 0.3 °C, oxygen between 5.0 to 8.0 mg / L, pH 7.6 ± 0.16 and total ammonium < 0.5 mg/L. A photoperiod of 12 h light and 12 h dark was used. The shrimp were fed with a commercial diet twice a day at 3% of their biomass. Shrimp were gradually acclimatized to 16 ppt and kept 15 days in observation before starting the experiment. Filters containing activated carbon were used to maintain an optimum seawater quality. Sixty percent of the water was replaced daily. At the end of the bioassay all materials were disinfected using granulated calcium hypochlorite at 1600 ppm and neutralized with sodium thiosulfate (Brenntag pacific Inc. Santa Fe Springs, CA 90670) at 872 ppm. The Infectious waste was sterilized before disposal.

**WSSV inoculum preparation.** The isolate of WSSV was used from a disease outbreak from Sonora, Mexico in 2008 (Son2008). The viral inoculum was prepared from frozen samples (−80 °C) of dying shrimp with WSSV positive diagnostic [45–46]. For this, 100 mg of gills from four individuals (25 mg each) were homogenized in 900 µL (1:10 ratio; mg/µL) of TN buffer (20 mM Tris-HCl, 400 mM NaCl, pH 7.4). The homogenized solution was centrifuged in two steps at 1800 and 3000*g* for 20 min, respectively, at 4 °C. The supernatant was recovered and filtered through a membrane filter (0.45 µm VWR^®^, Europe) [47]. This inoculum solution is referred to as the 1:10 dilution. The in vivo experiments were immediately initiated after preparing the inoculums. Simultaneously, uninfected shrimp or free WSSV were parallel-used under the same procedure as a negative control (WSSV-negative).

**Viral inoculum activation.** Two groups of 15 shrimp were inoculated with the solution obtained from infected shrimp as previously described. Then, the shrimp were transferred into 60 L rectangular aquariums. A third group (*n* = 15) was used as a control. Shrimp inoculation was performed by intramuscular injection (IM), using a 0.5 mL insulin syringe (BD Micro-FineTM) (31G × 6 mm), injecting 20 µL of 10^−1^ viral inoculum (original stock 1:10 p/v) to each shrimp in the fifth abdominal segment, whereas for the control group a TN sterilized buffer (20 mM Tris/HCl, 400 mM NaCl, pH 7.4) was used. The shrimp were fed commercial pellets three times a day. Every four hours, moribund organisms were collected and euthanized using liquid nitrogen, and subsequently stored at −80 °C for further analysis. WSSV was confirmed by endpoint polymerase chain reaction (PCR), following Koch's postulates.

**Minimum infectious dose determination.** The IM minimum lethal dose of WSSV to generate mortality as per os infection was determined simultaneously. Three replicates per treatment were used with six organisms (3.6 g ± 0.66 g) per aquarium. Before the viral challenge, shrimp were acclimatized for seven days under similar conditions. Then, shrimp were injected with 20 µL of a 10-fold serial dilution (10^−1^,10^−2^, 10^−4^, 10^−6^, 10^−8^, 10^−10^, 18 organisms per dilution) of WSSV inoculum (Son2008) stock 1:10 p/v. Shrimp were injected with virus-free gill homogenates, and TN buffer was used as control. The lethal dose 50% endpoint (LD_50_ mL^−1^) was calculated using the formula: log_10_ 50% endpoint dilution = − [(total number of animals died/number of animals inoculated per dilution) + 0.5] × log dilution factor [48]. To establish the per os WSSV infection time, five replicate aquaria with five shrimp (3.6 g ± 0.66 g) per tank were orally challenged. Before the infection per os, fasted shrimp for 24 hours were fed twice a day with infected ground tissue (≈10 biomass) [46]. Six hours after the last dose, the unconsumed infected tissue was removed, and aquarium water was replaced, per Thomas et al. [49]. Mortality was recorded to register the dose effectiveness of the inoculum (infected tissue) [50]. All collected shrimp (alive, dying, or dead) were cryo-frozen in liquid nitrogen (LN_2_) and stored at −80 °C for further analysis. All animal experimentation was supervised and authorized by the ethics committee of the institutional committee at UABC to comply with all the humanitarian protocols in handling to avoid animal suffering.

**Optimal dose of dsRNAvp28*.*** The optimal dose of the dsRNAvp28 (Genolution) was determined in a bioassay using different concentration doses. Five replicate aquaria with five juvenile shrimp (5.40 g ± 0.56 g) were used for the challenges. Organisms were acclimatized and fed as previously described. After seven days, 20 µL of WSSV inoculum (10^−6^ dilution) was applied (intramuscular injection) to each animal’s left side, simultaneously on the right side dsRNAvp28 was injected in doses of 0.5, 1.0, 2.0, and 3.0 µg/shrimp per group. A positive WSSV infection control without dsRNAvp28 treatment and a WSSV-free group were then injected with healthy tissue homogenate (20 µL) and 3.0 µg of dsRNAvp28/shrimp were included; see Table ST2 in Supporting Information File 1, material section.

**Administration of VLP-dsRNAvp28 by the oral cavity.** The inhibition efficacy of dsRNAvp28 to WSSV by oral route was evaluated using free dsRNAvp28 and VLP-dsRNAvp28 administered directly into the shrimp’s oral cavity. The procedure was standardized before the bioassay. In summary, 50 µL of TN solution containing 10% red food coloring (pigment red, McCormick4, USA) was administered through the oral cavity using an insulin syringe (BD Micro-FineTM) of 0.5 mL (31G × 6 mm). The distribution of the red-stained solution was observed with a stereoscopic microscope (Labomed, Model CZM6 Trinocular, Stereo Microscope) to determine the time and distribution of the product in the digestive tract of the shrimp.

After that, two sets of groups in four replicates with five shrimp each. In one of them, 6.0 µg (50 µL) of free dsRNAvp28 was administered, whereas in the second, 50 µL of VLP-dsRNAvp28 (6.0 µg of dsRNAvp28) was applied. After 18 hours both groups were challenged with WSSV by IM injection, with a dose of 10^−6^ Son2008 inoculum. (Herein “pellet feed” refers to when animals are fed with treatments, and “oral cavity” refers to when the VLPs treatment is given directly into the oral cavity through a needle to ensure intestinal functionality).

**Feed pellets with VLP-dsRNAvp28.** Two methods were used to prepare the pellet feed containing VLP-dsRNAvp28: first, coating the external surface of commercial pellets with the VLPs, and second, pulverizing the pellets, mixing the VLP’s with them, and reconstituting them (The details are described in Supporting Information File 1). In all experiments, to follow the standard procedures in bioassays with shrimp, each treatment had at least three replicates [10]. The pellets with VLP-dsRNAvp28 were coated with industrial grade fish oil or salmon fish oil (see details in Supporting Information File 1). Pellets with VLP-dsRNAvp28 prepared with commercial binders (Dry Oil^®^ and NutriKelp^®^) are described in Supporting Information File 1.

**Detection of WSSV by real-time quantitative PCR.** The real-time PCR (qPCR) for quantitation of WSSV was performed using DNA isolated from shrimp muscle tissue and TaqMan^®^ Fast Advanced Master Mix kit (Applied Biosystems, USA). Amplification reactions of 20 μL were prepared by mixing 23.33 ng of DNA, 0.3 μM of each primer, and 0.15 μM of TaqMan probe, and the qPCR was performed following Durand and Lightner [51] methodology. In summary, 2.0 min at 50 °C for uracil-*N*-glycosylase (UNG) activation; 10 min at 95 °C to activate AmpliTaq Fast DNA Polymerase and then, 40 cycles of 15 seconds at 95 °C and 1 min at 60 °C.

For the WSSV quantification, a standard curve was obtained with the plasmid DNA with the *vp664* gene of 69 bp [45,51] at a 1:10 dilution factor. The concentration range of the standard curve was 3.9 × 10^9^ to 3.9 × 10^4^ copies/ng. The ABI StepOnePlus v2.0 sequence detection system software (Applied Biosystems, USA) was used. Amplification reactions included all shrimp were analyzed (alive, dying and dead) from each experimental group.

The viral load of WSSV obtained by qPCR from three independent experiments was analyzed by comparing the average number of copies (copies/ng) of two replicates from the same shrimp of each group (*n* = 4–9 samples), plus their confidence interval.

**Statistical analysis**. For each treatment, the protection against WSSV after feeding with the antiviral therapy was evaluated through the survival and mean lethal time (LT_50_) [52]. A Log-Rank (Mantel–Cox) test was used to analyze the Kaplan–Meier survival curves generated with the GraphPad Prism version 5.01 software (San Diego California USA). In all cases, a value of *p <* 0.05 was considered significant. For the WSSV detection, an analysis of variance (ANOVA) was used to compare the average number of copies of WSSV and the average number of copies between treatments was analyzed with the Tukey’s test (*a* = 0.05). The Student's t-test was used to obtain significant differences (*t* – 95%) between treatments (alive vs dying/dead).

## Results

**Figure 1:**
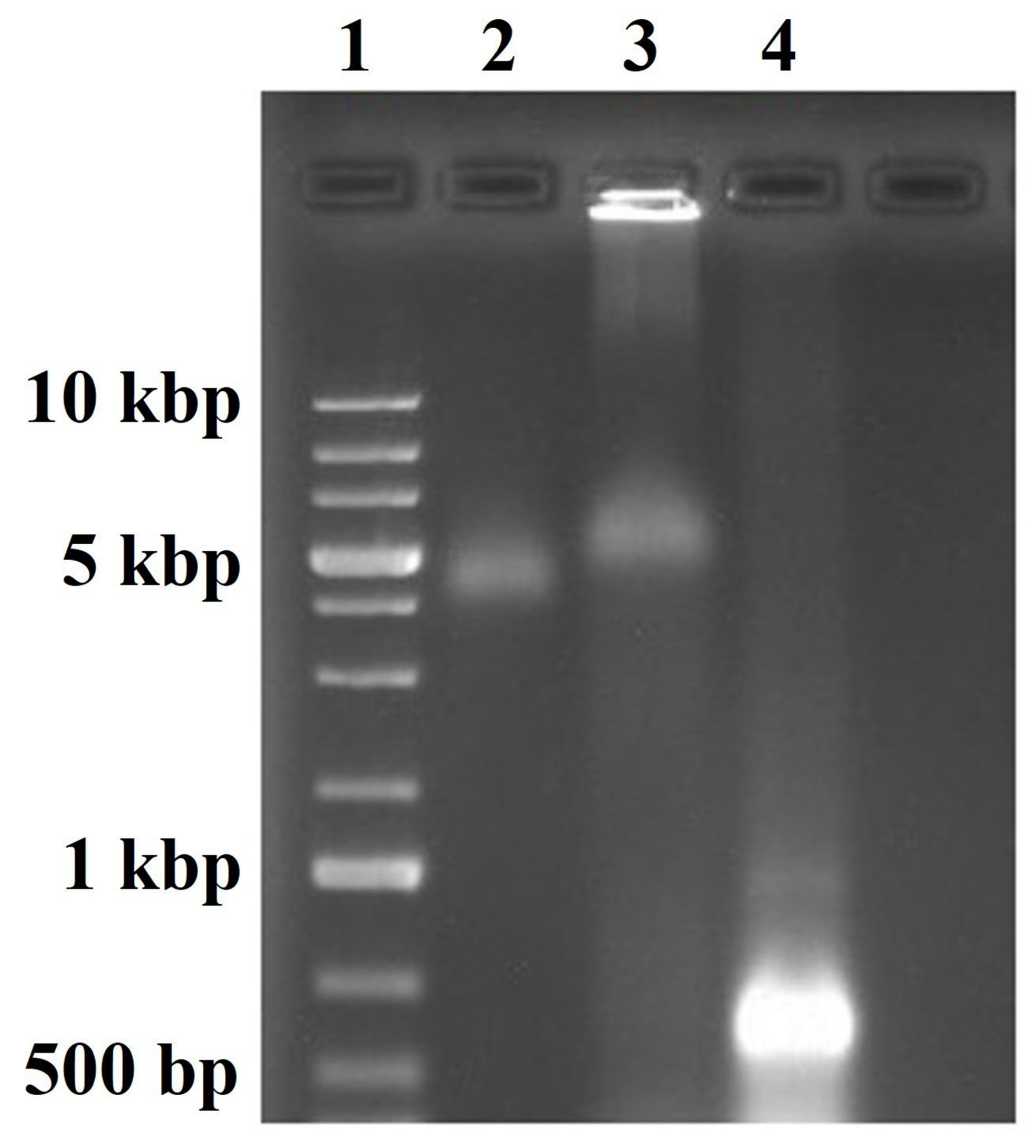
Analysis of the VLP-dsRNAvp28 assembly by electrophoresis mobility shift assay (EMSA) in a 1% native agarose gel. Lane 1 is the DNA ladder; 2: wild type CCMV; 3: self-assembly of dsRNA with CCMV CP; and lane 4: free dsRNA.

The dsRNA was efficiently encapsidated with CCMV CP using a mass ratio of 6:1 of CP/dsRNAvp28. The electrophoresis mobility shift assay (EMSA) of the assembly shows that most of the sample is close to the well, and a small sample portion migrated similarly to the wild type CCMV (lane 2 and 3, respectively, in Figure 1). In contrast, the free dsRNAvp28 ran faster (lane 4 in Figure 1) in comparison with the sample and wild type CCMV, as an indication of VLPs formation.

**Figure 2:**
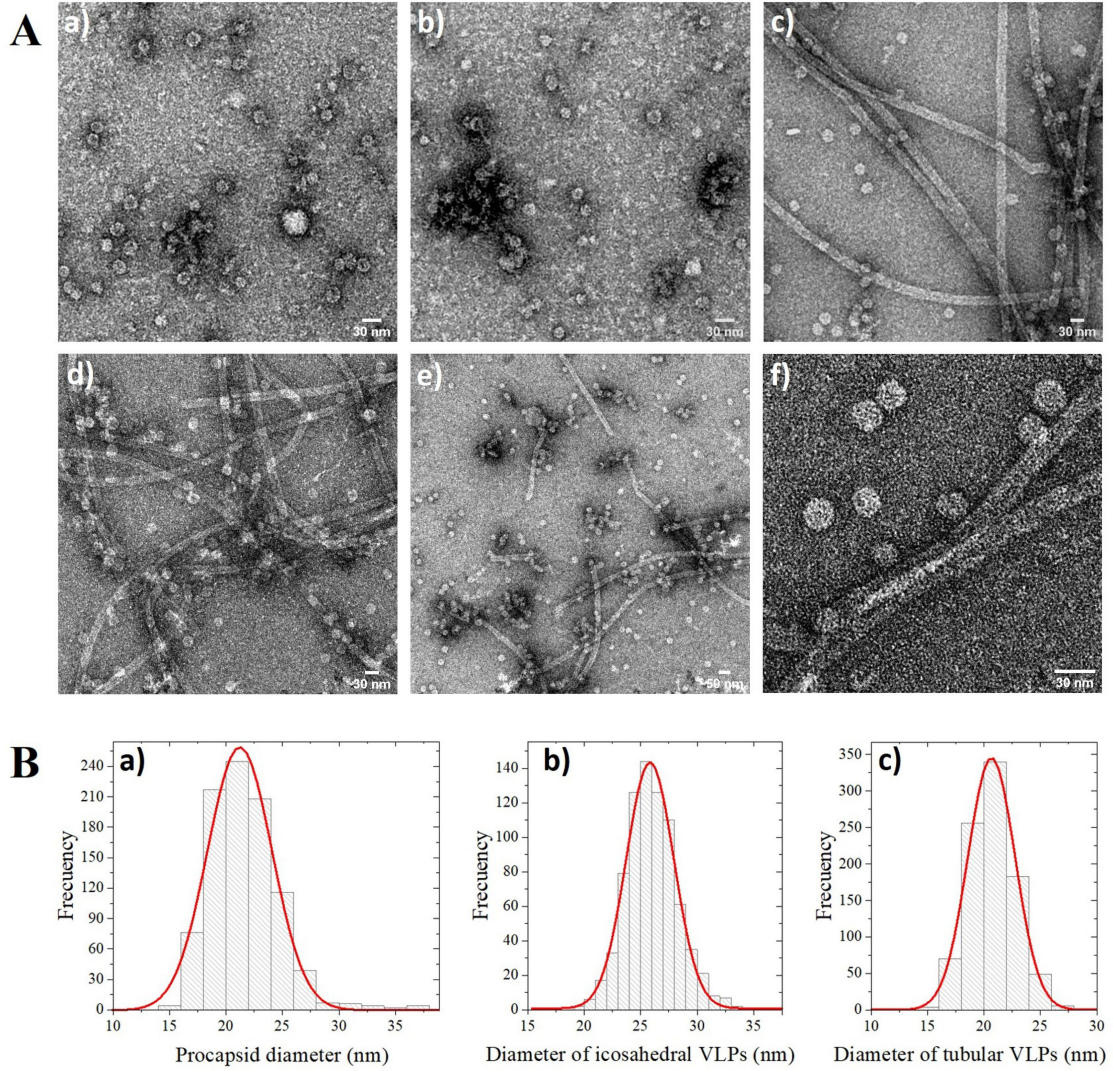
TEM micrographs of different stages of the assemblies of CCMV CP with dsRNAvp28. In section A, the images a) and b) correspond to the assembly in virus buffer; c) and d) are acidified assembly; e) and f) images correspond to the sample that was dialyzed again in assembly buffer. Section B shows the size distributions of the ensembles: a) diameter distribution of the procapsids with a mean diameter peak at 21 nm; b) diameters of the icosahedral VLP-dsRNAvp28 with a mean diameter peak at 26 nm and c) diameter of the tubular structures with a mean diameter peak at 21 nm.

The VLPs assembly at each stage was analyzed by transmission electron microscopy (TEM). The procapsids and CP-dsRNA complexes obtained after dialysis in assembly buffer (pH 7.2) can be observed in images a and b (Figure 2A). After the second dialysis in virus buffer (pH 4.5), well-defined VLPs were formed (Figure 2A, c, and d). Finally, the dialyzed sample is shown in Figure 2A, e, and f. The morphology of the VLPs is maintained after this last step of the assembly process. The VLPs synthesized had two types of morphologies: icosahedral capsids and large rods (Figure 2A, c to f). Also, aggregations of spherical capsid can be observed at the last VLP assembly step. The distribution of the procapsids diameter, icosahedral VLPs, and nanotubes is shown in Figure 2B. According to the Gaussian fit for each of the distributions, the average diameter of the procapsids, icosahedral VLPs, and the rods were 21, 26, and 21 nm, respectively.

**Figure 3:**
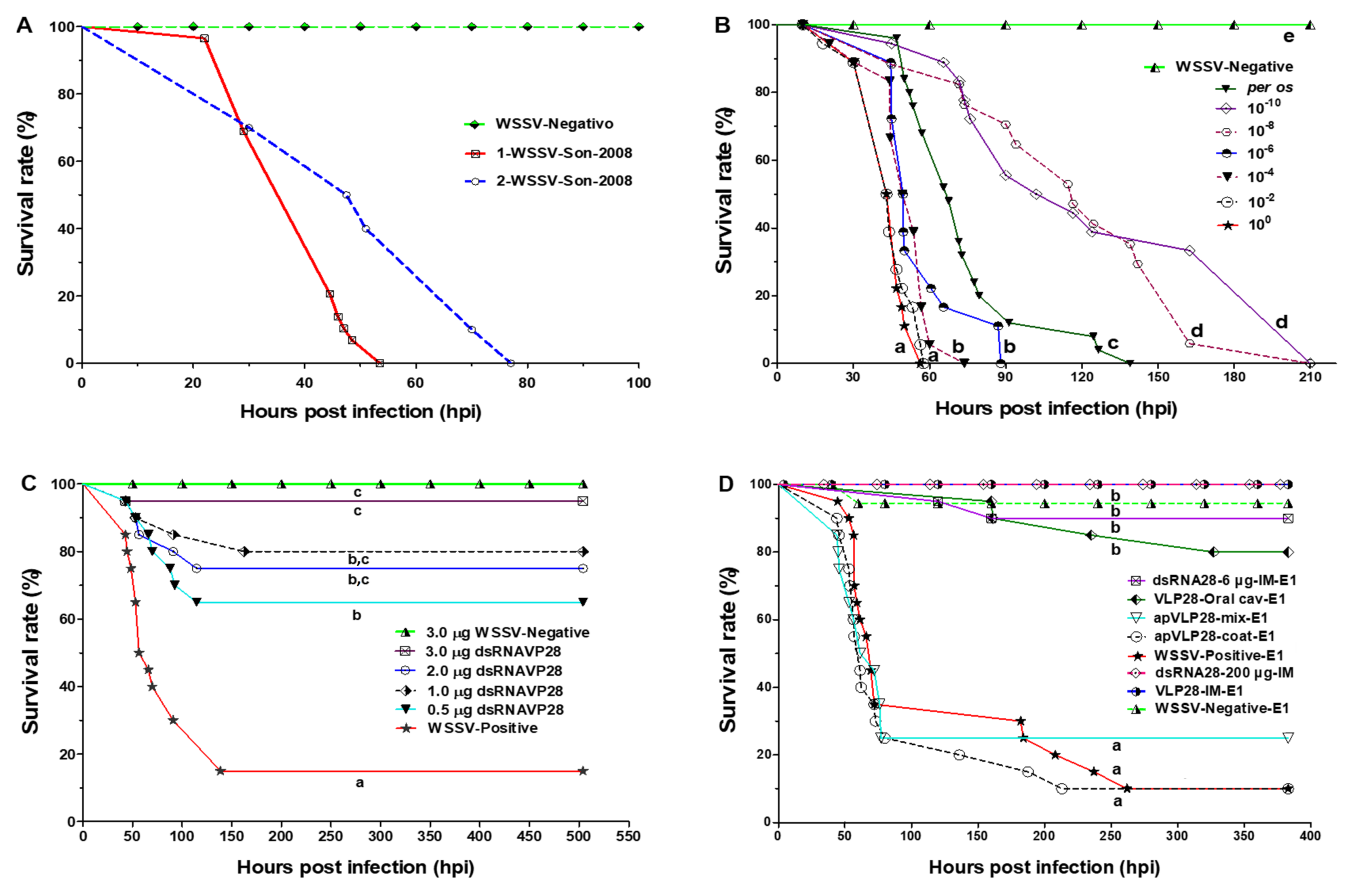
*P. vannamei* survival when exposed to WSSV and treatments. (A) IM inoculum activation in two consecutive experiments (1-WSSV-Son2008 and 2-WSSV-Son2008) (B) Per os infection with WSSV-Son2008 isolate to determine the LD_50_/mL (C) IM dsRNAvp28 at 3.0, 2.0, 1.0, and 0.5 µg, WSSV-Negative control received a 3.0 µg dose. The survival was evaluated up to 504 hpi (21 days). (D) Oral antiviral treatment with VLP-dsRNAvp28 (6 µg per shrimp) in the pellet with fish oil (industrial grade) as a binding agent. Different letters (a–d) in each experiment (B, C, and D) on the curves indicate significant differences (*p* < 0.0001) among treatments using the Log-rank (Mantel–Cox) Test and not the final absolute survival percentage. IM, intramuscular injection, see Table ST4 (Supporting Information File 1) for treatment abbreviature details.

During the WSSV viral inoculum activation, the symptoms' onset times and mortality occurred between 18 and 22 hours post-infection (hpi) (Figure 3A). At 22 hpi, the first death was detected. The minimum survival rates at 29 hpi, for the first inoculum reactivation, and 44.5 for the second (referred to as 1-WSSV-2008 and 2-WSSV-2008, respectively) were recorded. After 53.5 hpi, both for 1-WSSV-2008 and 77 hpi for 2-WSSV-2008, all shrimp were dead. Similarly, all infected shrimp from the control groups (WSSV-Positive) for the different treatments were dead. In contrast, 100% survival was obtained for the WSSV-Negative control groups (WSSV free).

**The minimum infectious dose of WSSV** resulted in significant differences (*p <* 0.001). The dilutions 10^−1^ and 10^−2^ gave 0% survival at 56.2 and 57.3 hpi, respectively. Whereas shrimp inoculated with the dilutions 10^−4^ and 10^−6^ showed complete mortality at 73.4 and 88.0 hpi, respectively. Moreover, the last group using 10^−8^ and 10^−10^ inoculum showed complete mortality at 162.3 and 210 hpi, respectively (Figure 3B).

The first deaths were recorded at 18 and 20.3 hpi for 10^2^ and 10^4^ dilutions, respectively. Regarding the per os infection, the first death was recorded at 47 hpi; all shrimp were dead at 139 hpi. The 10^−6^ dilution treatment resulted in an intermediate survival compared to the other dilution treatments, displaying a similar behavior as the per os infection. The calculated lethal dose at 50% endpoint dilution (LD_50_/mL) was 10^−6.5^. Therefore, the 10^−6^ dilution was used for the successive tests. The WSSV-Negative group showed 100% survival.

The WSSV-infected shrimp treated with different amounts (0.5, 1.0, 2.0, and 3.0 μg) of dsRNAvp28 through IM resulted in a significantly higher survival rate of >60% compared to the infected group without treatment (WSSV-Positive) with 15% survival in 21 days (Figure 3C). The high mortality of shrimp occurred between 70 and 100 hpi in all treatments. The survival curves resulted in a significant difference (*p* < 0.0001). When the WSSV-Negative (control non-infected) received 3.0 μg of dsRNAvp28/shrimp by IM, there was 100% shrimp survival. In comparison, the infected group treated with 3.0 μg of dsRNAvp28/shrimp showed only one death at 43 hpi (95% survival) during the 21 days of the experiment. As a result of the dose-response, 3.0 μg/shrimp was chosen as the subsequent dose for the IM treatments.

Different results were obtained with treated shrimp fed with pellets carrying the VLP-dsRNAvp28. When pellets were coated with VLP-dsRNAvp28 mixed with fish oil (ApVLP28-coat-E1) there was a 10% survival. Whereas those fed with ApVLP28-mix-E1 resulted in 25% survival (Table ST4, Exp. 1 in Supporting Information File 1) up to 384 hpi. However, the positive group resulted in 100% survival. The control group from the dsRNA28-6 µg-IM-E1 treatment achieved a 90% survival compared to the VLP28-IM-E1 group, with 100% survival (Figure 3D).

When the VLPs were administered via the oral cavity (VLP28-oral cav-E1), an 80% survival was obtained. Moreover, the group of shrimp that were given an IM dose of 200 µg of free dsRNAvp28 and infected with WSSV (dsRNA28-200 µg-IM) all survived up to the end of the experiment, 16 days post-infection (dpi), without showing any abnormal symptoms or behavior observable due to high dose of dsRNAvp28 (Figure 3D).

**Figure 4:**
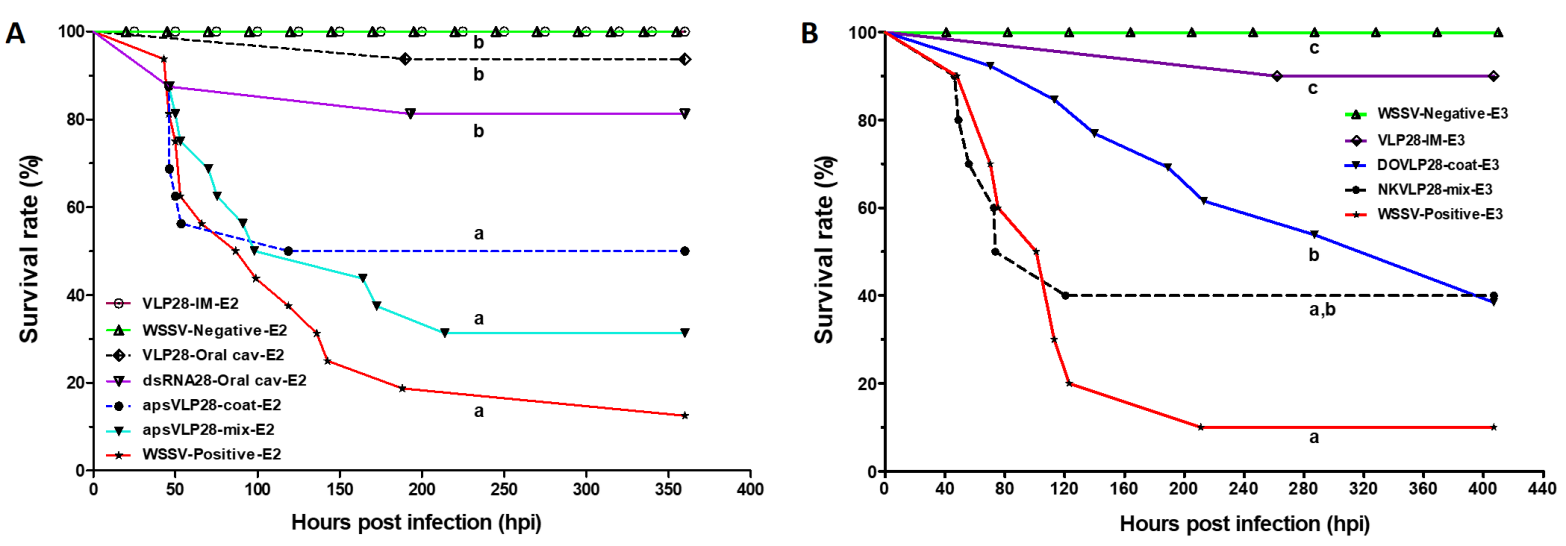
Cumulative survival curves of *P. vannamei* infected with WSSV and provided with VLPs antiviral treatment. (A) Pellets with VLP-dsRNAvp28 and covered with salmon oil (apsVLP28-coat or mixed). Administration by oral cavity (VLP28-oral cav-E2, and dsRNA28-oral cav-E2) means that the antiviral was given with a syringe right into the oral cavity. Note that these groups had high survival (93.75% and 81.25%) up to 350 hpi or 15 days. (B) Pellets with VLP-dsRNAvp28 prepared with commercial binders (Dry Oil^®^ and NutriKelp^®^). The bioassay was ended 17 days post-treatment (or 400 hpi). Different letters (a–c) on the curves indicate significant differences (*p* < 0.0001) between treatments with Log-rank (Mantel–Cox), not the final absolute survival percentage. IM, intramuscular injection, see Table ST4 in Supporting Information File 1 for treatment abbreviature details.

When VLP-dsRNAvp28 was used to coat pellets with salmon oil alone (ApsVLP28-coat-E2) or mixed (ApsVLP28-mix-E2), low survival was observed (50 and 31.25%, respectively) (Figure 4A). Simultaneously, the VLP28-IM-E2 group resulted in 100% survival until the end of the experiment (15 dpi). The groups treated by oral cavity (VLP28-oral-cav-E2) or naked dsRNA28 (dsRNA28-oral cav-E2) had 93.75 and 81.25% survival. In contrast, the WSSV-Positive-E2 showed a 12.5% survival rate until the end of the experiment (360 hpi, 15 days). Moreover, no significant differences between this group and those treated orally with ApsVLP28-coat-E2 and ApsVLP28-mix-E2 were found; whereas the WSSV-Negative-E2 treatment had a 100% survival rate.

While the Dry Oil^®^ binder (DOVLP28-coat) and NutriKelp^®^ binder (NKVLP28-mix) were used to incorporate the VLPs, a 38.5 and 40% survival rate was obtained, respectively. Whereas the control groups VLP28-IM-E3, WWSV-Negative-E3, and WSSV-Positive-E3 had a survival rate of 90, 100, and 10%, respectively. The cumulative survival curves of treatments with pellets VLP-dsRNAvp28 prepared using commercial binders are shown in Figure 4B.

The analysis of qPCR data showed that the viral load decreases significantly (*p <* 0.05) in WSSV-infected shrimp survival when orally treated with VLP-dsRNAvp28 (VLP28-mix and VLP28-coat), compared to positive controls (WSSV-Positive). However, similar results were obtained for shrimp-fed pellets prepared with different binders (fish oil and commercial binders). Organisms treated with VLPs by IM (VLP28-IM) or oral cavity (VLP28-Oral-cav) therapy were WSSV negative in more than 90% (15–17 dpi). After 60 dpi, the organisms treated IM with VLP-dsRNAvp28 had a slight degree of infection. Shrimp treated by oral antiviral therapy, with coated and mixed pellets (VLP28-coat and VLP28-mix) and collected dying or dead, resulted in higher viral load concentrations compared to those collected alive (Table 1). However, at the end of the experiment those shrimp collected alive were positive for WSSV, but with a slight degree of infection. The WSSV-Negative controls were free of virus.

**Table 1:** WSSV copies in shrimp abdominal tissue by real-time quantitative PCR (qPCR). Average copies of WSSV in ng^−1^ and SD values of shrimp treated with coated and mixed pellets using industrial-grade fish oil (ap), salmon fish oil (aps), and commercial binders (DO and NK).

treatment	live		dying/dead	

	average^a^	SD	average^a^	SD

apVLP28-mix^b^	2.39 × 10^10^	3.10 × 10^10^	3.027 × 10^10^	2.93 × 10^10^
apVLP28-coat^b^	9.36 × 10^4^	3.89 × 10^4^	1.23 × 10^10^	5.82 × 10^9^
apsVLP28-mix^c^	2.32 × 10^4^	2.71 × 10^4^	7.39 × 10^9^	1.07 × 10^10^
apsVLP28-coat^c^	2.01 × 10^4^	1.48 × 10^4^	4.79 × 10^9^	4.04 × 10^9^
DOVLP28-mix	1.11 × 10^4^	6.85 × 10^3^	6.33 × 10^9^	6.76 × 10^9^
NKVLP28-coat	7.87 × 10^3^	7.75 × 10^3^	7.91 × 10^8^	6.98 × 10^8^
WSSV-positive			1.30 × 10^10^	2.60 × 10^10^

^a^WSSV copies [ng^−1^]; ^b^ap = industrial grade fish oil; ^c^aps = salmon oil.

## Discussion

CCMV VLPs containing dsRNA were successfully synthesized to silence the WSSV VP28 protein expression. Here we used a 6:1 mass ratio of capsid protein to dsRNA, according to previous works for the encapsidation of ssRNA [42] and siRNA [32]. To our knowledge, this is the first report showing a long dsRNA encapsidation using a plant virus capsid protein.

The analysis by EMSA showed that the VLPs that self-assemble migrate differently than the free dsRNAi (Figure 1, lane 4). After dialysis in assembly buffer (pH 7.2), the sample analysis by TEM shows the spherical procapsids formation and CP-dsRNAi complexes (Figure 2A, images a and b). The Gaussian fit size distribution of the spherical procapsids gave an average diameter of 21.2 nm and corresponded to capsids with triangulation number *T* = 2. The sample’s dialysis at pH 7.2 favors the electrostatic interactions between CCMV CP-dsRNA to form procapsids and CP-RNA complexes [42]. The dsRNA negative charges can be neutralized by the positive N-terminal protein in these procapsids [42]. However, procapsids are not suitable for any treatment because they do not efficiently protect their cargo. The dsRNA in the procapsids may be degraded by nucleases [42,53]. The appropriate synthesis of VLP-dsRNAvp28 was only obtained after the sample was acidified by dialysis in virus buffer (pH 4.5). After acidification no more aberrant and complex capsids were observed (Figure 2A, images c and d). The low pH promotes protein–protein interactions and allows the stable forms of VLPs [54]. The TEM micrographs revealed two types of VLPs shapes: the icosahedral and the long tubular structures. The individual VLPs with a spherical (icosahedral) shape are likely to have few ssRNA molecules, due to the low contamination of the RNAi stock with ssRNA of 563 nts (according to the company that synthesized the RNA). The icosahedral VLPs are not empty, because in assembly buffer the CCMV CP form capsids only when anionic molecules are present.

On the other hand, the long tubular structures result from the experimental conditions during the VLPs formation. Due to the isoelectric point of the capsid protein (pH ≈ 4.8), the protein charge can easily modify the capsid protein dimers’ spontaneous curvature, leading to the formation of tubular structures [55–56]. Also, the dsRNAvp28 is a long dsRNA with a persistence length of around 60 nm [57–58] that could be favorable for tube formation. The interaction of a rigid and quasi-long dsRNA molecule with the capsid protein dimers enables the elongated tubular structure formation under these experimental conditions [59].

The spherical VLPs have an average diameter of 25.8 nm corresponding to capsids with a triangulation number *T* = 3, similar to the wild type (wt) CCMV [53]. Whereas in the nanotubular VLPs, a diameter of 21.7 nm is revealed. In this work, the nanotubular length was not determined because the tubular synthesized VLPs are very long, and some are curved, making the measurement difficult. The correlation of the TEM and EMSA results suggests that the band in the agarose gel migrated slightly less than the wild type CCMV corresponds to, in multiple icosahedral capsids and short tubes with dsRNA. Similar results have been obtained with long ssRNA [42]. In contrast, the band that is close to the well corresponds to the long nanotubes. The individual icosahedral VLPs are not possible to visualize in the gel electrophoresis due to their low concentration in the sample. Most of dsRNAvp28 is self-assembled into long nanotubular VLPs, and similar results have been reported with dsDNA [60].

The CCMV has been reported to be biocompatible in mammals, testing the wild-type virus in mice [32,61]. However, there were no studies to demonstrated non-toxicity in other species such as crustaceans and fish. Therefore, before performing the bioassays with VLP-dsRNAvp28, this study evaluated the toxicity of wt CCMV in healthy shrimp. The bioassay was carried out for three weeks, and the shrimp showed no symptoms of any disease or apparent abnormality when treated by IM (up to 20 µg of CCMV per shrimp). Higher doses of dsRNAvp28 (200 µg) per WSSV infected shrimp by IM injection were also evaluated, showing no adverse effects or evident disease (Figure 4A).

The biocompatibility of CCMV in shrimp is of great commercial significance. The biocompatibility of CCMV suggests a broad potential to develop treatments for disease control in aquatic organisms and mammals.

Plant virus-based VLPs, in general, are particularly advantageous in aquaculture and medicine because they are biocompatible, biodegradable, and do not infect mammals [32,62] or marine organisms. To date, CCMV has shown the ability to be distributed widely in mouse organs and tissues using different administration routes [61]. Also, the CCMV VLPs are resistant to enzyme degradation through the digestive tract [32–34]. It is to be kept in mind that possibly the shrimp’s virus, in contrast to CCMV VLP’s, needs specific receptors to be internalized in the shrimp cells. For these reasons, CCMV VLPs show quite an advantage over the VLPs derived from the shrimp virus.

The mortality rate of shrimp inoculated with WSSV is dose-dependent [52,63–64]. Dose dependency can be grouped in three virulence levels, according to the dilutions used: high 10^−1^–10^−2^ (45–43.5 hpi), medium 10^−4^–10^−6^ (51.4–49.5 hpi) and low 10^−8^–10^−10^ (116.5–109.3 hpi). It is important to note that similar mortality behavior was observed between dilution 10^−6^ and infection per os. The median lethal dose obtained here (10^−6.5^ LD_50_/mL) is consistent with previous reports [46,52]. In our experiments, the cumulative mortality of 100% for the 10^−6^ (LD_50_/mL) dose was at 88 hpi, and the median lethal time LT_50_ was 49.58 hpi.

The amount of inoculum orally ingested was estimated to be more than that of IM injection, because only a small proportion of the virus inoculated orally can infect shrimp [46]. However, we observed that challenged shrimp did not consume all the macerated infected tissue offered. Then, by inoculation per os, ≈10% of infected tissue biomass was used (for two days). It registered an accumulated mortality rate of 92% at 124.5 hpi and 100% at 139 hpi. In our study, the median survival time was 67.7 hpi. However, even if the results are consistent, the infection by IM injection is recommended in challenge bioassays, allowing greater viral dose control, compared to infection per os where it is difficult to calculate the consumption of infected shrimp tissue [65].

By IM injection, the dsRNAvp28 resulted in a great protective efficacy in *P. vannamei* against WSSV infection. Experimental results indicate that a minimum dose of 0.5 µg/shrimp is enough to protect up to 65% of the population against the virus. The maximum dose used in the present work was 3.0 µg/shrimp with 95% protection at 504 hpi (21 days). These evaluated doses are lower than those previously reported from 5.5 μg doses [66] up to 31 μg of dsRNA/shrimp [23]. This work has demonstrated the efficacy of the dose, and the sequence of the dsRNA used. According to our results, and considering possible losses by dispersing the VLP-dsRNAvp28 in the water or inside the shrimp, a maximum dose of 6 µg of dsRNA/shrimp as a single dose can be considered for oral administration.

The treatments using salmon fish oil to adhere the CCMV VLP28 to the feed pellet showed an increase in shrimp survival up to 50% (ApsVLP28-coat). On the other hand, therapy with VLP-dsRNAvp28 taken orally was more effective than when merely present in the feed as a coating. Taking the VLP-dsRNA28 orally assures capsid functionality by protecting the dsRNA structure. Administering VLP capsids inside the feed resulted in increased shrimp survival after challenged with the WSSV and treated per os. The survival rates obtained were 38.5% and 40% with DOVLP28-coat and NKVLP28-mix, respectively. Although the percentage with DOVLP28-mix is lower than NKVLP28-coat, the mortality was higher with the last treatment, reaching a 50% mortality rate at 73.6 hpi compared to 287 hpi that reached 53.8% mortality rate with DOVLP28-mix. This protection is significantly higher compared to the first results using fish oil. Both treatments using commercial binders indicate that it is possible to administer it in the pellets. However, it is crucial to state that pellets usually undergo pelleting or extrusion, damaging the VLPs. Therefore, further studies should be on how this can be administered in the pellets at industrial levels.

Other studies by IM injection of chemically modified chitosan nanoparticles loaded with anti-VP28 RNA [20] and antisense plasmid constructs for VP28 [24] have shown protection of 95% and 90%, respectively. However, in all these treatments the shrimp exposed to WSSV finally died at 14 dpi. To date, only two works have reported the use of VLPs with dsRNAvp28 against WSSV. In both cases, the VLPs were synthesized from viruses that infect shrimp. One was with the macrobrachium rosenbergii nodavirus (MrNv) [66], whereas the second was with the infectious hypodermal and hematopoietic necrosis virus (IHHNV) [31]; both studies showed a survival rate of 44.5 and 40% by IM injection (6 μg of dsRNAvp28 per shrimp), respectively. Here, we were able to obtain similar results when VLP-dsRNAvp28 was administered per os. However, we experiment with the same dose of VLP-dsRNAvp28 (6.0 µg/shrimp), equivalent to the same dilution of WSSV to infect them. But the bioassay was finished at 17 dpi.

However, one should not rule out possible differences in the shrimp origin line (genetics, immunology), feeding factors, manipulation (stress), the pathogenicity of the used WSSV isolate, and the infective dose, among others. By IM injection with the CCMV VLP-dsRNAvp28, we found survival rates of up to 100% with 17 dpi and up to 50% survival rates at 60 dpi using one single dose of 6 µg. In contrast with IM administration reports, we showed a good survival rate by oral antiviral therapy. It is important to note that our results show practically 100% protection through IM injection. Xie et al. [27] considered that the main difficulty in applying RNAi in shrimp in vivo is its intracellular release. Although naked dsRNA can penetrate cell membranes when injected locally, it is rapidly degraded by plasma nucleases.

The treated organisms with VLP-dsRNAvp28 by oral cavity obtained an 86% survival rate. However, during the oral cavity application treatment (VLP28-oral cav-3 and dsRNA28-oral cav), some shrimp regurgitated part of the treatment, so the efficacy by this route was 86.8 and 81.2% survival, respectively (experiment E2). Although oral cavity and IM application showed a high survival compared to the administration of the VLP-dsRNAvp28 in pellet, it could indicate that VLPs: 1) were lost in the water by pellet detachment; 2) were not ingested by shrimp; 3) shrimp enzymes degraded it; or 4) a high concentration of VLPs was lost in feces. We hypothesized that an adequate amount is not being absorbed, since the observed survival rate does not exceed 50%. Thus, the problem is not the treatment itself but the dose that finally reaches the shrimp tissues. An investigation will be conducted testing higher doses.

Oral antiviral treatment in aquatic organisms is not straightforward because of the enormous challenges of breaking the water barrier. Therefore, for therapy or vaccine, it is essential to maintain, before ingestion, the compound’s stability and the adherence to the pellets. When shrimp eat the pellet they have the peculiar tendency to fragment it (due to its size, and to food selectivity for palatability, hardness). This differs from fish, who swallow the whole pellet. Therefore, a considerable amount of VLP-dsRNAvp28 can be lost in the water while the shrimp is feeding.

The experiments presented were performed using different shrimp sizes from 3.6 ± 0.7 to 17.7 ± 2.7 g. However, no size effect could be detected on the amount of dsRNAvp28 administered IM and orally. The efficacy of dsRNAvp28 by IM from 3.0 to 6.0 µg per organism, was effective in small and large shrimp, indicating the possibility that doses used are higher than required.

The efficacy of CCMV VLP-dsRNAvp28 to protect WSSV infected shrimp was verified by qPCR. Viremia was reduced in orally treated organisms. Therefore, oral administration should be considered effective as antiviral therapy before viral infection, since extra doses will be necessary. (But keep in mind that infected shrimp will stop eating from three to four days after initial infection, so oral therapy at that point cannot cure them). The qPCR data analysis indicates that VLP-dsRNAvp28 by oral therapy reduces the mortality rate by reducing the WSSV infection.

Mejía-Ruiz et al. [28] reported that antiviral protection provided by a single IM administration of dsRNAvp28 is short-lived, 10 to 20 days post-treatment (dpt), with 63% and 87% mortality rate, respectively, being gradually lost after 30 dpt, Also Witteveldt et al. [67] observed that viral protection in *P. monodon* was reduced 21 days after administering orally VP28 expressed in bacteria as an antiviral treatment. Furthermore, Ufaz et al. [20] showed that the protective effect of treatment remains active at least two weeks after viral exposure. In shrimp farms usually, the WSSV is not detected until dead organisms are perceived, making it impossible to determine precisely the time of infection. However, it might be possible to protect neighbor ponds or farms once the onset of a local viremia is detected nearby.

We hypothesize that antiviral therapy based on CCMV VLP-dsRNAvp28 with a single dose by oral administration cannot exceed one month of protection. According to the survival results, the IM injection up to two months protection could be achieved. For this reason, the antiviral therapy would be based mainly on preventive therapy or at the first signs of infection, through continuous prophylaxis during the period of the shrimp culture. By this means, the risk of crop losses before a potential outbreak occurs could be avoided. Once shrimp are infected by the WSSV, they will stop eating within 18 to 24 hpi, so at that point, oral administration is no longer possible.

In this work, we have shown that VLPs derived from the CCMV have a high potential as a vehicle for RNAi delivery. Likewise, the brome mosaic virus (BMV) VLPs-dsRNAvp28 show similar results to those of the CCMV (data not shown). Furthermore, these VLPs can be chemically modified with a peptide or using protein engineering, to express on its external surface to better recognize (target) the WSSV infected cells increasing the antiviral therapy efficiency.

Because new viral outbreaks are the primary threat to aquaculture production, innovative biosecurity measures to limit production losses are essential [68–69]. Biosecurity programs do not always reduce the incidence of outbreaks in areas where the WSSV is prevalent in natural carriers [2]. Thus, current prevention strategies do not eradicate the virus. It is imperative to find prevention that works. Vaccines or antiviral therapies to effectively control or eliminate these outbreaks should be a priority in further investigations. The Government and private sector should work together to develop strategies to protect the profitability of the aquaculture sector [70].

## Conclusion

This work represents the first study of long dsRNAs encapsidation using plant virus capsid proteins, such as CCMV, for WSSV treatment in shrimp. Our results indicate that intramuscular injection treatment revealed a survival rate of nearly 100%, while a 90% survival is shown by oral cavity administration using CCMV VLP-dsRNAvp28 in shrimp infected with WSSV. However, using the CCMV VLPs orally administered in feed pellets resulted in a survival rate of 40%.

Our preliminary results shown here with CCMV VLP-dsRNAvp28 offers adequate protection against WSSV. Although the therapy proves effective protection, reinforcement to protect the organisms during a culture season or when an outbreak begins to occur in neighboring ponds or farms also can be applied.

We report the different strategies that provide a significant advance in methods for the delivery of therapeutic molecules. The antiviral therapy here presented could be applied, with further research, to other aquatic species or even terrestrial organisms, or within nanomedicine applications.

## Supporting Information

File 1 Tables of detailed experimental assays and methods to prepare the pellet feed containing VLP-dsRNAvp28.
