# Supplementary material for: Antiviral therapy in shrimp through plant virus VLP containing VP28 dsRNA against WSSV
Source: Beilstein J Org Chem. 2021 Jun 1;17:1360–73. doi: 10.3762/bjoc.17.95 (PMC8182676; doi:10.3762/bjoc.17.95)
Supplement: File 1 — Tables of detailed experimental assays and methods to prepare the pellet feed containing VLP-dsRNAvp28. [file Beilstein_J_Org_Chem-17-1360-s001.pdf]

BEILSTEIN JOURNAL OF ORGANIC CHEMISTRY

Supporting Information

for

Antiviral therapy in shrimp through plant virus VLP containing VP28 dsRNA against WSSV

Santiago Ramos-Carreño, Ivone Giffard-Mena, Jose N. Zamudio-Ocadiz, Alfredo Nuñez-Rivera, Ricardo Valencia-Yañez, Jaime Ruiz-Garcia, Maria Teresa Viana and Ruben D. Cadena-Nava

Beilstein J. Org. Chem. **2021,** *17,* 1360–1373. doi:10.3762/bjoc.17.95

Tables of detailed experimental assays and methods to prepare the pellet feed containing VLP-dsRNAvp28

License and Terms: This is a supporting information file under the terms of the Creative Commons Attribution License (https://creativecommons.org/licenses/by/4.0). Please note that the reuse, redistribution and reproduction in particular requires that the author(s) and source are credited and that individual graphics may be subject to special legal provisions.
The license is subject to the *Beilstein Journal of Organic Chemistry* terms and conditions: (https://www.beilstein-journals.org/bjoc/terms)

Table ST1. Partial sequence of the VP28 gene of the main structural protein of WSSV isolated from Mexico (GenBank: EU931451.1).

```
ATGGATCTTTCTTTCACTCTTTCGGTCGTGTCGGCCATCCTCGCCATCACTGCTGTGATTGCTGTATTTA
TTGTGATTTTTAGGTATCACAACACTGTGACCAAGACCATCGAAACCCACACAGACAATATCGAGACAAA
CATGGATGAAAACCTCCGCATTCCTGTGACTGCTGAGGTTGGATCAGGCTACTTCAAGATGACTGATGTG
TCCTTTGACAGCGACACCTTGGGCAAAATCAAGATCCGCAATGGAAAGTCTGATGCACAGATGAAGGAAG
AAGATGCGGATCTTGTCATCACTCCCGTGGAGGGCCGAGCACTCGAAGTGACTGTGGGGCAGAATCTCAC
CTTTGAGGGAACATTCAAGGTGTGGAACAACACATCAAGAAAGATCAACATCACTGGTATGCAGATGGTG
CCAAAGATTAACCCATCAAAGGCCTTTGTCGGTAGCTCCAACACCTCCTCCTTCACCCCCGTCTCTATTG
ATGAGGATGAAGTTGGCACCTTTGTGTGTGGTACCACCTTTGGCGCACCAATTGCAGCTACCGCCGGTGG
AAA
```

Table ST2. Design of the challenge to evaluate the efficacy and optimal dose of dsRNA*vp28*.

Group[1]	Treatment scheme	No. of shrimp[2]
WSSV-Positive	+WSSV infected shrimp	4 × 5
3.0 µg WSSV-Negative	-WSSV free shrimp 3.0 µg dsRNA*vp28* IM	4 × 5
0.5 µg dsRNA*vp28*	+0.5 µg dsRNA*vp28* IM	4 × 5
1.0 µg dsRNA*vp28*	+1.0 µg dsRNA*vp28* IM	4 × 5
2.0 µg dsRNA*vp28*	+2.0 µg dsRNA*vp28* IM	4 × 5
3.0 µg dsRNA*vp28*	+3.0 µg dsRNA*vp28* IM	4 × 5

[1]Indicates the abbreviature for each treatment
(+) WSSV-Positive shrimp with a dose of 10^{-6}; (-) WSSV-Negative or non-infected shrimp. [2]Indicates the number of replicates × number of shrimps in each replicate.

Methods to prepare the pellet feed containing VLP-dsRNAvp28.

Method I. Pellets of similar size (Natural Force, VIMIFOS. México) were coated with 0.5 µg of VLP-dsRNAvp28 each. VLP-dsRNAvp28 were applied coating the pellets with a thin layer of fish oil before drying at room temperature [1]. The feed was dried at 28 °C for 15 min and stored at 4 °C. The pellets were offered twice a day (2.0 µg/shrimp/day), 2 pellets at 09:00 h. and two at 16:00 h.) for three continuous days. After 48 hour feeding period, the shrimp were challenged with the WSSV.

Method II. The pellets were pulverized using a sterile porcelain mortar until a fine powder was obtained and mixed with 3% (w/v) NutriKelp® binder and fish oil in hot water (90 °C). After the blend was cooled to ca. 35 ° C, the solution of VLP-dsRNAvp28 and 5% fish oil was added. The proportion recipe for the preparation of the pellets is shown in Table ST3. Afterward, the mixture was pelleted pressing through a 50 mL syringe without a needle (Terumo® 50 mL) to make long pellets (≈ 10–12 cm) and dried in an incubator (VWR Incubator F Air 2.3 CF, USA) at 28 °C for 40 min. The pellets were cut into pieces of ≈ 2 to 2.5 mm long. Feed prepared with VLPs was stored at 4 °C for subsequent doses. The pellets obtained (10 g) were weighed and divided by the number of organisms to be treated (*n* = 20). The ration for each shrimp (0.5 g) was divided into six portions, to offer twice a day. The feed was administered at 2 µg of dsRNAi/shrimp daily, as described in the Method I section, giving a total of 6 µg of dsRNA/shrimp over a period of three days. In both cases the organisms were starved for 24 hours [2] before giving them the VLP-dsRNAvp28 treatment.

Pellets with VLP-dsRNAvp28 coated with industrial grade fish oil. Industrial grade fish oil was used to coat the pellets with VLPs following the Method I and II. The treatments were performed using shrimp (10.0 g ± 1.1 g) acclimated for 15 days at a salinity of 16 ppt. The experimental conditions are detailed in Table ST4 (Experiment 1).

Pellet with VLP-dsRNAvp28 coated with salmon fish oil. The salmon fish oil (Carlson Labs, Wild Norwegian Cod Liver Oil) was used to coat the pellets with VLPs following the Method I and II. Seven treatments were performed, each with four replicates with four shrimp (14.5 g ± 2.1 g) each (n = 16) in 16 ppt saline water. The details are described in Table ST4, Experiment 2.

Pellet with VLP-dsRNAvp28 prepared with commercial binders.

a). Dry Oil®. The pellet feeds were prepared as described in Method I with slight changes. Dry Oil® (DO, Innova-Codemet SA de CV, Mexico) was used as a binder to coat pellets with VLPs, following the manufacturer's instructions. The pellets were directly submerged in a solution of Dry Oil in TN buffer at 30 °C containing the VLP-dsRNAvp28. The Table ST4 describe in details of this treatment, Experiment 3. Due to the characteristics of this product, it was only used to coat the pellets.

b). NutriKelp®. Pellets with VLPs using NutriKelp® (NK, NutriKelp Algas & Bioderivados Marinos, Mexico) as a binder. The procedure to make this food is described in "Method II." Experimental details are in Table ST4 (Experiment 3). The Nutrikelp has an alginate base; it was only used to remake the pellet and not to coat them.

Table ST3. Recipe to prepare pellets mixed with VLPdsRNA*vp28* to feed shrimps.

Ingredient	Amount	To observe
Powdered pellet	8.0 g/ Xg[a]	The formation of fine grains
Natural grenetine hydrated		3% total in the mix
Fish oil	0.5 mL	
VLPdsRNA*vp28** (0.12 µg/µL)	1.0 mL	1X PBS[b]
Pellet mixed with VLPdsRNA*vp28*	≈ 10 g	Make pellet

[a]The amount used may vary depending on the shrimp weight, to administrate twice a day, approximately 3% daily biomass for 3.3 g shrimp.
[b]Make pellet with 50 mL syringe without needle, and dry at 28–29 °C × 40 min, then cut in pieces 2 to 2.5 mm long.

Table ST4. Oral feeding challenges with VLPdsRNA*vp28* in *Penaeus vannamei*.

**Experiment**	**Group***	**Treatment scheme**	**No. of shrimp in group**[a]
**Exp. 1.**	WSSV-Positive-E1	+WSSV infected shrimp	4 × 5
	WSSV-Negative-E1	-WSSV free shrimp	4 × 5
	VLP28-IM-E1	+Control VLPdsRNA*vp28* IM	4 × 5
	ApVLP28-mix-E1	+VLPdsRNA*vp28*/Mixpell	4 × 5
	ApVLP28-coat-E1	+VLPdsRNA*vp28*/coatpell	4 × 5
	VLP28-oral cav-E1	+VLPdsRNA*vp28*/oral cavity	4 × 5
	dsRNA28-200 µg-IM	+Naked dsRNA*vp28*/200 µg IM	2 × 6
	dsRNA28- 6 µg IM-E1	+Naked dsRNA*vp28* -IM	4 × 5
**Exp. 2.**	WSSV-Positive-E2	+ WSSV infected shrimp	4 × 4
	WSSV-Negative-E2	-WSSV free shrimp	4 × 4

	VLP28-IM-E2	+Control VLPdsRNA*vp28* IM	4 × 4
	ApsVLP28-mix-E2	+VLPdsRNA*vp28*/Mixpell	4 × 4
	ApsVLP28-coat-E2	+VLPdsRNA*vp28*/coatpell	4 × 4
	VLP28-Oral cav-E2	+VLPdsRNA*vp28*/oral cavity	4 × 4
	dsRNA28-Oral cav-E2	+Naked dsRNA*vp28* /Oral cavity	4 × 4
	WSSV-PositiveE3	+WSSV infected shrimp	1 × 10
	WSSV-Negative-E3	-WSSV free shrimp	2 × 15
**Exp. 3.**	VLP28-IM-E3	+Control VLPdsRNA*vp28* IM	3 × 10
	NKVLP28-mix-E3	+VLPdsRNA*vp28*/Mixpell	3 × 10
	DOVLP28-coat-E3	+VLPdsRNA*vp28*/coatpell	3 × 10

[*]=Treatment key. E = Experiment number.
"+"=WSSV-Positive (dose 10^{-6}); "-" WSSV-Negative, Mixpell = mixed pellet, coatpell = coated pellet.
Shrimps treated with VLPdsRNAvp28 or naked dsRNAvp28 received a dose of 6 µg dsRNA, unless otherwise specified. [a]Indicates the number of replicates × number of shrimps in each replicate.

Exp. 1. Pellets with VLPdsRNA*vp28* prepared with fish oil (Ap) (industrial grade)

Exp. 2. Pellet with VLPdsRNA*vp28* coated with salmon fish oil (Aps)

Exp. 3. Pellet with VLPdsRNA*vp28* prepared with commercial binders (DO/NK= (Dry Oil® and NutriKelp®, respectively).

References

1. Jha, R. K.; Xu, Z. R.; Bai, S. J.; Sun, J. Y.; Li, W. F.; Shen, J. *Fish Shellfish Immunol.* **2007**, *22*, 295–307. doi:10.1016/j.fsi.2006.04.006
2. Ning, J. F.; Zhu, W.; Xu, J. P.; Zheng, C. Y.; Meng, X. L. *Vaccine* **2009,** *27*, 1127–1135. doi:10.1016/j.vaccine.2008.11.075
